# The Rho Termination Factor of *Clostridium botulinum* Contains a Prion-Like Domain with a Highly Amyloidogenic Core

**DOI:** 10.3389/fmicb.2015.01516

**Published:** 2016-01-07

**Authors:** Irantzu Pallarès, Valentin Iglesias, Salvador Ventura

**Affiliations:** Institut de Biotecnologia i Biomedicina and Departament de Bioquìmica i Biologia Molecular, Universitat Autònoma de BarcelonaBarcelona, Spain

**Keywords:** prion, bacteria, *Clostridium*, protein aggregation, amyloid

## Abstract

Prion-like proteins can switch between a soluble intrinsically disordered conformation and a highly ordered amyloid assembly. This conformational promiscuity is encoded in specific sequence regions, known as prion domains (PrDs). Prions are best known as the causative factors of neurological diseases in mammals. However, bioinformatics analyses reveal that proteins bearing PrDs are present in all kingdoms of life, including bacteria, thus supporting the idea that they serve conserved beneficial cellular functions. Despite the proportion of predicted prion-like proteins in bacterial proteomes is generally low, pathogenic species seem to have a higher prionic load, suggesting that these malleable proteins may favor pathogenic traits. In the present work, we performed a stringent computational analysis of the *Clostridium botulinum* pathogen proteome in the search for prion-like proteins. A total of 54 candidates were predicted for this anaerobic bacterium, including the transcription termination Rho factor. This RNA-binding protein has been shown to play a crucial role in bacterial adaptation to changing environments. We show here that the predicted disordered PrD domain of this RNA-binding protein contains an inner, highly polar, asparagine-rich short sequence able to spontaneously self-assemble into amyloid-like structures, bearing thus the potential to induce a Rho factor conformational switch that might rewire gene expression in response to environmental conditions.

## Introduction

Amyloid forming proteins are found in all kingdoms of life, from Bacteria to Animalia (Fowler et al., 2007; Eichner and Radford, 2011; Sanchez de Groot et al., 2012). Although amyloid formation is associated with the onset of debilitating human disorders such as Alzheimer’s, or Parkinson’s (Maries et al., 2003; Stohr et al., 2012), the amyloid fold is also exploited for evolutionary selected biological functions by diverse species, including humans (Chiti and Dobson, 2006; Furukawa and Nukina, 2013). Prions are a particular type of amyloids that can switch between soluble and self-templating aggregated states. In the so-called functional prions, this property is used to perform important functions, acting as epigenetic elements and supporting beneficial roles in cell physiology (Newby and Lindquist, 2013).

The conformational duality of prion-like proteins resides in structurally independent, low complexity, prion-forming domains (PrDs), usually enriched in asparagine (N) and glutamine (Q) residues (Dorsman et al., 2002; Fandrich and Dobson, 2002; Halfmann et al., 2011). This composition endorses the domains with intrinsic structural disorder, which enables self-assembly without a requirement for conformational unfolding (Fuxreiter, 2012; Malinovska et al., 2013). Much research has gone in the recent years into uncovering how prion propensities are encoded in protein sequences (Alberti et al., 2009; Toombs et al., 2010; MacLea et al., 2015; Sabate et al., 2015a) and several algorithms exploit this knowledge to identify new putative prion proteins (Toombs et al., 2012; Espinosa Angarica et al., 2013, 2014; Lancaster et al., 2014; Sabate et al., 2015b; Zambrano et al., 2015). The high-throughput analysis of proteomes using these programs has led to the identification of thousands of new potential prion-like proteins in organisms belonging to all taxonomic subdivisions (Espinosa Angarica et al., 2013). The results show that, in general, the number of prions per genome is low, less than 1% of the complete proteome (Michelitsch and Weissman, 2000; Harrison and Gerstein, 2003; Espinosa Angarica et al., 2013). Ontology analysis indicates that PrD-containing proteins are associated with a great variety of physiological functions, supporting prion-like proteins acting as beneficial elements for organisms.

In a previous work, we have used our algorithm PrionScan to analyze 839 different bacteria proteomes, detecting 2200 putative prions in these organisms (Espinosa Angarica et al., 2013, 2014). Interestingly, we found a special enrichment in proteins containing PrDs in pathogenic bacteria (Espinosa Angarica et al., 2013). A significant number of these proteins are DNA or RNA binding proteins (Iglesias et al., 2015), which might be involved in host induced bacteria gene expression plasticity, recapitulating the response of yeast transcription factors with prion-like properties in front of environmental fluctuations (Alberti et al., 2009; Malinovska et al., 2013; Newby and Lindquist, 2013).

PrionScan identifies PrDs on the basis of their amino acid compositional similitude to *bona fide* yeast prions, which results in a very fast algorithm useful to scan very large databases, as those corresponding to a complete taxon (Espinosa Angarica et al., 2014). However, this speed comes at the cost of a lower specificity in the predictions, when compared with competing algorithms like PAPA (Toombs et al., 2012) and pWALTZ (Sabate et al., 2015b). PAPA exploits the compositional bias of PrDs to identify these domains in protein sequences using a experimentally derived amino acid prion propensity scale (Toombs et al., 2010), whereas pWALTZ implements a totally different concept, since it assumes that it is the presence and potency of specific short amyloid-prone sequences that occur within intrinsically disordered Q/N-rich regions that account for prion induction (Sabate et al., 2015b).

Here, we combined PAPA and pWALTZ algorithms to get highly specific PrDs predictions in the proteome of *Clostridium botulinum* (*C. botulinum*). This bacterium is widely spread in the environment, with reservoirs both in soil and water sediments and is a well-known pathogen that affects animals and humans worldwide (Espelund and Klaveness, 2014). This approach led us to the identification of 54 putative prion proteins. Among them, it outstands the transcription termination factor Rho (Rho) (Richardson, 1990, 1996; Boudvillain et al., 2013). We show here that its predicted PrD contains a highly polar, N-rich, short sequence stretch able to form amyloid-like fibrils, which might endorse this RNA-binding protein with the ability to shift from soluble to aggregated states in order to modulate its functionality.

## Materials and Methods

### Prion Forming Domains identification in Bacteria

The *C. botulinum* E1 str. ‘BoNT E Beluga’ proteome dataset was downloaded from Uniprot (release 2015_05) and scanned for PrDs using PAPA (Toombs et al., 2012) with the default parameters, which includes the disorder prediction algorithm FoldIndex (Prilusky et al., 2005). From the initial 3678 proteins in the proteome, 63 prion-like candidates were identified. Their putative prion forming domains were further evaluated with pWALTZ (Sabate et al., 2015b) using the default parameters to identify those domains containing a putative amyloid core, which resulted in 54 final positive predictions.

### *Clostridium botulinum* PrD Peptide Preparation

A peptide with the sequence NNNNSNFNNNSNNNSSFNNSN, corresponding to the predicted amyloid core in the PrD of *C. botulinum* Rho factor, was purchased from CASLO ApS. Stock solutions were prepared at 5 mM in DMSO and stored at -80°C. For analysis, the peptide was diluted to 25, 50, and 100 μM in PBS buffer.

### Aggregation Assays

Aggregation of initial soluble species was monitored by following the transition from non-aggregated to aggregated states by measuring light scattering at 360 nm in 25, 50, and 100 μM peptide samples at 25°C. Light scattering changes were evaluated for samples incubated during 4, 48, and 120 h.

### Binding to Amyloid Dyes

The binding of 25 μM of Thioflavin-T (Th-T) to Rho peptide was recorded using a Cary Eclipse Spectrofluorometer (Varian, Palo Alto, CA, USA) with an excitation wavelength of 440 nm and emission range from 460 to 600 nm at 25°C in PBS buffer. Spectra were recorded after 2 min of equilibration, and solutions without peptide were used as negative controls. Excitation and emission slit widths of 10 nm were used. For the staining assays with Thioflavin-S (Th-S), Rho peptide aggregates were incubated for 1 h in the presence of 125 μM of dye. After centrifugation (14000 ×*g* for 5 min), the precipitated fraction was washed twice with PBS and placed on a microscope slide and sealed. Images of Rho peptide fibrils bound to Th-S were obtained at 40-fold magnification under UV light or using phase contrast in Leica fluorescence microscope (Leica DMRB, Heidelberg, Germany).

Congo red (CR) interaction with Rho peptide aggregates was tested using a Cary100 UV/Vis spectrophotometer (Varian, Palo Alto, CA, USA) by recording the absorbance spectra from 400 to 675 nm using a matched pair of quartz cuvettes of 1 cm optical length placed in a thermostated cell holder at 25°C. Final CR and peptide concentrations were 5 μM in PBS buffer. In order to detect the typical amyloid band at ∼541 nm, differential CR spectra in the presence and absence of peptide were recorded.

### Bis-ANS Binding

Binding of 4,4′-bis (1-anilinonaphthalene 8-sulphonat) (bis-ANS) to Rho peptide was evaluated by registering bis-ANS fluorescence between 400 and 700 nm after excitation at 370 nm on a Cary Eclipse Spectrofluorometer (Varian, Palo Alto, CA, USA). Spectra were recorded at 25°C in PBS buffer, final peptide and dye concentrations were 10 and 1 μM, respectively. Excitation and emission slit widths of 10 nm were used.

### Aggregation Kinetics and Seeding Assays

Rho peptide aggregation was monitored by quantification of the changes in relative Th-T fluorescence at 475 nm when exciting at 440 nm along time. In the seeding assay, a solution of 0.1% (w/w) preformed fibrils was added at the beginning of the reaction. All experiments were carried out in PBS buffer under agitation (∼750 rpm with micro-stir bars) at 25°C with an initial soluble peptide concentration of 100 μM.

### Secondary Structure Determination

ATR FT-IR spectroscopy analysis of Rho peptide aggregates was performed using a Bruker Tensor FT-IR Spectrometer (Bruker Optics, Berlin, Germany) with a Golden Gate MKII ATR accessory. Each spectrum consists of 16 independent scans, measured at spectral resolution of 1 cm^-1^. Infrared spectra between 1725 and 1575 cm^-1^ were fitted through overlapping Gaussian curves, and the amplitude, and area for each Gaussian function were calculated employing the non-linear peak-fitting program (PeakFit package, Systat Software, San Jose, CA, USA).

### Transmission Electron Microscopy (TEM)

For negative staining, samples of Rho peptide incubated at 25°C for 4, 48, and 120 h were placed onto carbon-coated copper grids and left to stand for 5 min. The grids were washed with distilled water and stained with 2% (w/v) uranyl acetate for 2 min. Micrographs were recorded in a JEM-1400 (JEOL, Japan) transmission electron microscope (TEM) operated at 80-kV accelerating voltage.

## Results

### Identifying Prion-Like Domains on the Pathogenic Bacteria *Clostridium botulinum*

Recent bioinformatics screenings revealed multiple prion candidates in bacteria, especially in pathogenic species (Espinosa Angarica et al., 2013; Iglesias et al., 2015). In light of these data, we focused here on the Gram-positive, anaerobic bacterium *C. botulinum*, given its involvement in a number of pathological processes (Swaminathan and Eswaramoorthy, 2000; Kumaran et al., 2009; Rossetto et al., 2014). The analysis of the 3678 protein sequences in *C. botulinum* proteome was initially performed with PAPA (Toombs et al., 2012) and further refined with pWALTZ (Sabate et al., 2015b). Both PAPA and pWALTZ algorithms were trained on top of yeast prions; however, they are based on radically different concepts, a suitable composition of the PrD and the presence of an amyloid core embedded in it, respectively. This ensures that sequences that pass the two thresholds should have properties resembling previously verified yeast prions. According to their respective scores, 54 proteins, corresponding to 1.5% of the proteome, were identified as containing PrDs in *C. botulinum* (Supplementary Table S1). Ontology analysis indicates that the putative prion-like dataset is enriched in biological processes related to the cell wall dynamics. However, we also found proteins relevant in bacterial processes such as invasion, virulence and nucleotide metabolism (Supplementary Table S1).

We analyzed the role of the structural Pfam domains linked to the detected *C. botulinum* PrD-containing proteins. As expected, the biggest cluster of Pfam families is associated with cell wall dynamics, with 19 out of the 41 annotated putative prions having a cell wall binding repetition domain. Among the proteins in that cluster we can find a glycosyl transferase (C5UUW9), which is a glycan synthesis effector and a clear example of proteins involved in cell wall rearrangement, with a structure combining two different functional domains, one glucoamylase domain and two glycotransferase domains. The cell shape protein MreC (C5UR99), is another relevant protein in that cluster, which is thought to couple the internal bacterial cytoskeleton to the extracellular cell wall synthesizing complexes; interestingly, it is a protein that associates with penicillin-binding proteins and guides the insertion of newly synthetized cell wall precursors (Divakaruni et al., 2005; Tavares et al., 2015). Yet another protein in this subset is Brachyurin (C5UXB1), a cell-wall associated protein that contains two N-cadherin domains in its structure, suggesting a role in cell–cell contact, adhesion and biofilm formation (Anantharaman and Aravind, 2010). The second most abundant group of Pfam domain families is associated to invasion and virulence processes. This group includes proteins associated with encapsulation, sporulation and toxins. CotH (C5UUU1) and the spore cortex-lytic enzyme (C5U536) are proteins required either for spore coat formation (Zilhao et al., 1999) or for spore germination, thus facilitating *C. botulinum* aerial growth, surface attachment and pathogenesis. We also find a L,D-transpeptidase (C5UVDO), which cross-links peptidoglycan in presence of antibiotic drugs that block regular effectors (Biarrotte-Sorin et al., 2006; Magnet et al., 2007) allowing the bacteria to overcome classical β-lactams antibiotic blockage. We highlight in this cluster the presence of the Botulinum neurotoxin non-toxic-non-hemagglutinin component (NTNH). The neurotoxin complex is composed of NTNH, the toxin BoNT, hemagglutinin (HA) and associated subcomponent proteins and RNAs (Wren, 1991). It has been proposed that NTNH confers protection against the harsh conditions the toxin faces in the digestive tract (Sugawara et al., 2014). The third group contains proteins with domains involved in nucleotide binding, such the Transcription termination factor Rho (C5URV5) involved in transcription regulation and the Ribonucleoside-diphosphate reductase (C5UTH8) that is implicated in DNA replication. Other relevant putative prion-like proteins that cannot be clustered in the former groups but merit attention are StbA (C5UUD6), a putative Hsp70 family chaperone which has been seen to stabilize plasmids and control their number in *Escherichia coli* (*E. coli*) (Bork et al., 1992; Guynet et al., 2011) and a putative ggdef domain protein (C5UR68), with two relevant functional domains, a tetratricopeptide domain, involved in scaffold formation to mediate protein interactions and the assembly of multiprotein complexes and a GGDEF domain related with the synthesis of cyclic di-GMP and involved in the regulation of processes such as biofilm formation, motility and cell differentiation.

### Rho Factor Exhibits a Predicted PrD Containing a Putative N-rich Amyloid Core

Because many of the prion-like polypeptides identified in eukaryotes are RNA binding proteins (King et al., 2012; Kim et al., 2013), we focused our attention in the transcription termination factor Rho (Rho). Rho is required for the factor-dependent transcription termination by an RNA polymerase in prokaryotes and is essential for the viability of the cell (Richardson, 1996; Cardinale et al., 2008; Washburn and Gottesman, 2011; Krishna Leela et al., 2013). Recent studies indicate that besides being a housekeeping gene, Rho can function as a gene regulator and participates in the control of prophage maintenance in bacterial genomes (Boudvillain et al., 2013; Menouni et al., 2013). Accordingly, it plays a critical role in determining what proteins are present in the cell, in what amounts and thus modulating the organism’s phenotype.

**FIGURE 1:**
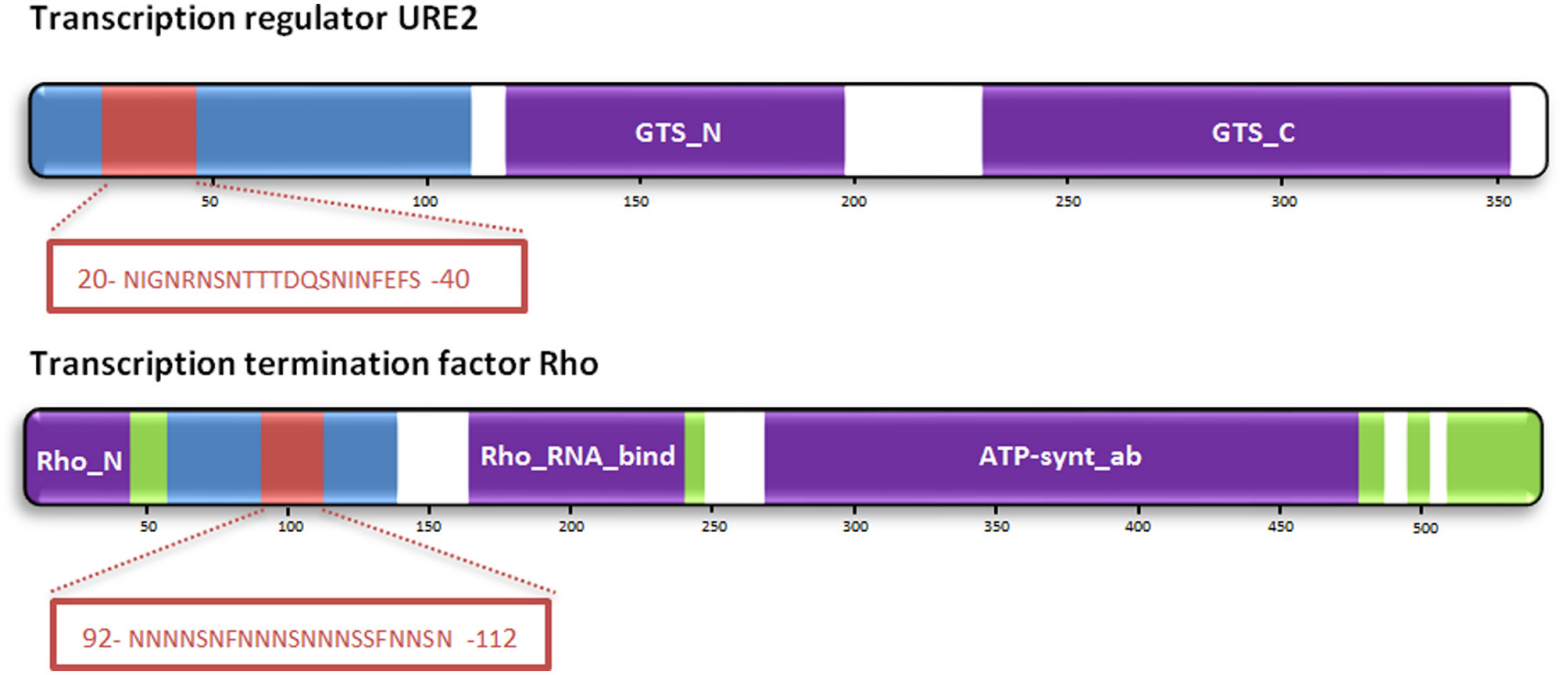
**Prion domains and amyloid cores as predicted in the sequences of Rho from *Clostridium botulinum* and the prion protein URE2 from *Saccharomyces cerevisiae*.** Identified Pfam domains, disordered regions, predicted PrDs and amyloid cores are shown in violet, green, blue, and red, respectively.

PAPA predicts an 80 residues long PrD close to the Rho factor N-terminus, which resides in a longer intrinsically disordered region, as predicted with FoldIndex (Prilusky et al., 2005) (**Figure 1**). pWALTZ predicts the presence of three overlapping 21 residues long amyloid stretches comprising residues 90–110, 92–112, and 93–113 inside the identified Rho PrD (**Figure 1**). When we analyzed the location of structured, unstructured and PrD regions in Rho factor, we found that, overall, its topology resembles that observed in certain *bona fide* yeast prions, like Ure2p (**Figure 1**). Globular domains in prion-like proteins are responsible for their biological function. The Rho factor consists of six identical subunits, each containing three functional domains. The RNA binding site has been localized to the N-terminal portion of the protein, the ATP binding site is located in the central portion of the primary sequence, and subunit interaction sites have been proposed to reside in the C-terminal region (Geiselmann et al., 1993; Bogden et al., 1999). The interaction of Rho with RNA is critical to all the activities of the protein. Thus RNA binding is required to activate the RNA-dependent ATPase activity of Rho. The predicted PrD and the RNA binding domain are contiguous in Rho, a topology that is also found in many eukaryotic prion-like proteins (King et al., 2012; Espinosa Angarica et al., 2013; Malinovska et al., 2013; Navarro et al., 2015).

The widely accepted “amyloid-stretch” hypothesis proposes that the amyloid potential of amyloidogenic proteins resides in short, highly amyloidogenic regions that act by nucleating the aggregation reaction (Ventura et al., 2004; Esteras-Chopo et al., 2005). We have recently proposed that this view also applies for prion-like proteins, explaining why all known prions adopt amyloid conformations in their propagative state (Sabate et al., 2015a). In order to assess if this is the case of Rho factor, we experimentally characterized the predicted central amyloid core of the prion domain (cPrD) using a synthetic peptide corresponding to sequence 92-NNNNSNFNNNSNNNSSFNNSN-112, with a 67% N content. Despite pWALTZ, which is specially intended to analyze PrDs, predicts that this N-rich sequence would endorse the surrounding PrD with significant amyloidogenic potential, well-contrasted aggregation predictors like AGGRESCAN (Conchillo-Solé et al., 2007), TANGO (Fernandez-Escamilla et al., 2004) or FoldAmyloid (Garbuzynskiy et al., 2010) fail to predict any aggregation-prone region in this peptide and, indeed, they predict it to be soluble.

### Rho cPrD Forms β-sheet Enriched Aggregates

**FIGURE 2:**
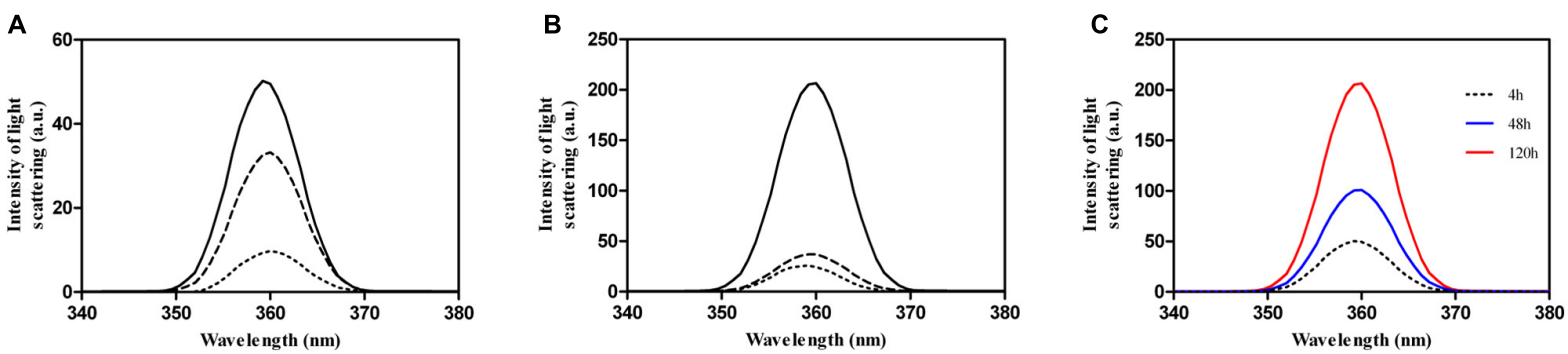
**Aggregation of Rho cPrD as a function of the concentration and the incubation time.** Aggregation changes were monitored by light scattering at different Rho cPrD concentrations 25 (doted line), 50 (dashed line), and 100 μM (solid line) incubated at 25°C during **(A)** 4 h, **(B)** 120 h. The scattering at 4, 48, and 120 h for the 100 μM peptide solution is shown in **(C)**.

As a first step to experimentally characterize the selected cPrD we analyzed its *in vitro* aggregation properties. Rho cPrD was incubated at 25, 50, and 100 μM at 25°C for 4, 48, and 120 h and aggregation from its initially soluble state was evaluated using synchronous light scattering (**Figure 2**). A concentration dependent scattering signal is observed after 4 h. However, the signal corresponding to the 25 and 50 μM solutions does not evolve significantly with time, whereas the scattering signal of the 100 μM peptide solution steadily increases to attain a maximum after 120 h (**Figure 2C**). Accordingly, unless otherwise indicated, all subsequent experiments were performed with the peptide at a concentration of 100 μM.

**FIGURE 3:**
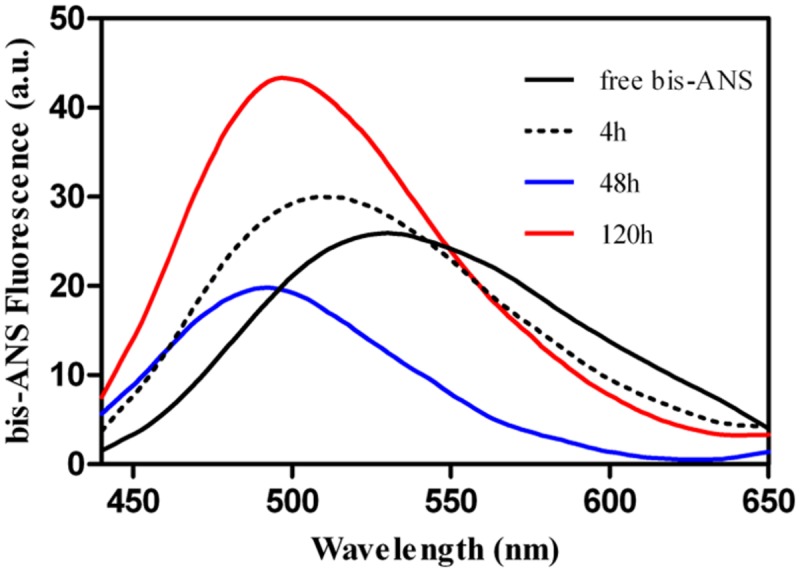
**Binding of bis-ANS to 100 μM Rho cPrD as a function of the incubation time at 25°C**.

For most amyloids, the self-assembly reaction depends on the formation of intra-chain hydrophobic clusters (Hills and Brooks, 2007). However, Rho cPrD is a highly polar peptide, with less than 10% of its residues being hydrophobic. We explored the presence of exposed hydrophobic clusters in the aggregates formed by Rho cPrD at different times by measuring their binding to bis-ANS (**Figure 3**), a dye that increases its fluorescence emission upon interaction with these regions (Gohlke, 1972; de Groot et al., 2007; Zhou et al., 2012). The bis-ANS fluorescence emission maximum blue-shifts from 530 nm, in the absence of peptide, to 509 nm in the presence of the peptide after 4 h. This spectral change is even more pronounced after 48 h, even if the global intensity decreases. Bis-ANS fluorescence emission attains a maximum at 120 h, with its spectral maximum blue-shifted to 490 nm. These data clearly indicate that the two phenylalanine (F) residues in Rho cPrD play an important role on its aggregation reaction, leading to the formation of strong hydrophobic patches in the final aggregates.

**FIGURE 4:**
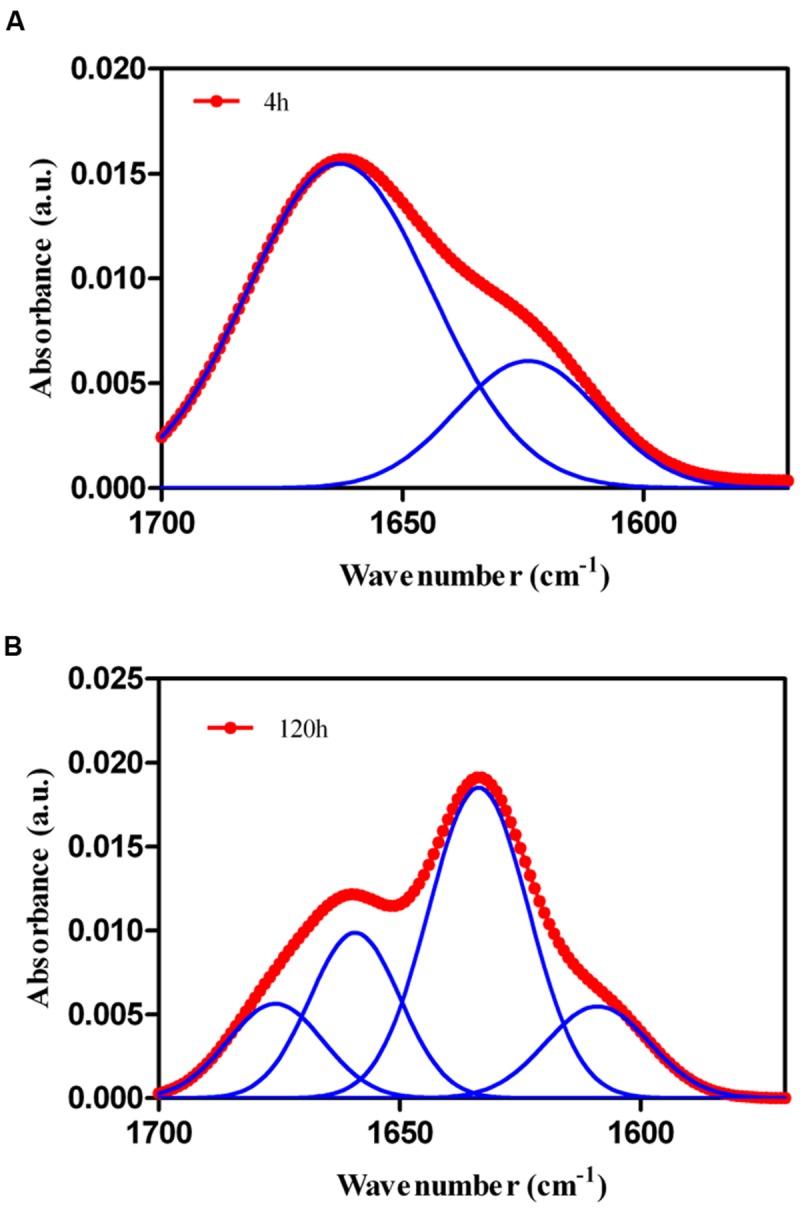
**Conformational properties of 100 μM Rho cPrD as a function of the incubation time at 25°C during **(A)** 4 h, **(B)** 48 h.** Secondary structure was determined by ATR FT-IR. Red circles show the sum of individual spectral components, blue lines represent different secondary structure signals arising from Gaussian deconvolution.

The aggregation of proteins into amyloid fibrils results in the formation of intermolecular β–sheets (Nelson et al., 2005). To get insights into the secondary structure content of the assemblies formed by Rho cPrD, we analyzed the amide I region of the FTIR spectrum (1700–1600 cm^-1^) (**Figure 4**). This region corresponds to the absorption of the carbonyl peptide bond group of the protein main chain and is a sensitive marker of the protein secondary structure. Examination of the secondary structure of Rho cPrD peptide by deconvolved FTIR spectra allow us to assign the individual secondary structure elements and their relative contribution to the main absorbance signal at the beginning (4 h) and ending (120 h) of the aggregation reaction (**Figure 4**; **Table 1**). After 4 h of incubation the spectrum of Rho cPrD is dominated by a band at 1663 cm^-1^, corresponding to disordered structures, accounting for 74% of the total area. However, the presence of an inter-molecular β–sheet component at 1624 cm^-1^ is already observable at this time point. At the end of the reaction (120 h), the FTIR spectrum of Rho cPrD is dominated by a band at 1633 cm^-1^ attributable to β–sheet conformations. At this stage, the low frequency β–sheet components at 1607 and 1633 cm^-1^ together with the high frequency β–sheet component at 1676 cm^-1^ account for 77% of the total area, with disordered conformations contributing only 23% of the signal. These spectral properties are compatible with the assembly of Rho cPrD into a highly β–sheet enriched amyloid-like structure.

**Table 1:** Assignment of secondary structure components of Rho cPrD peptide in the amide I region of the FTIR spectra.

Rho PrD 4 h			Rho PrD 120 h		
Band (cm^-1^)	Area (%)	Secondary structure	Band (cm^-1^)	Area (%)	Secondary structure
1624	26	β-sheet	1607	15	β-sheet
1663	74	Loops/turns	1633	48	β-sheet
			1661	23	Loops/turns
			1676	14	β-sheet

### Rho cPrD Self-Assembles into Amyloid Fibrils

**FIGURE 5:**
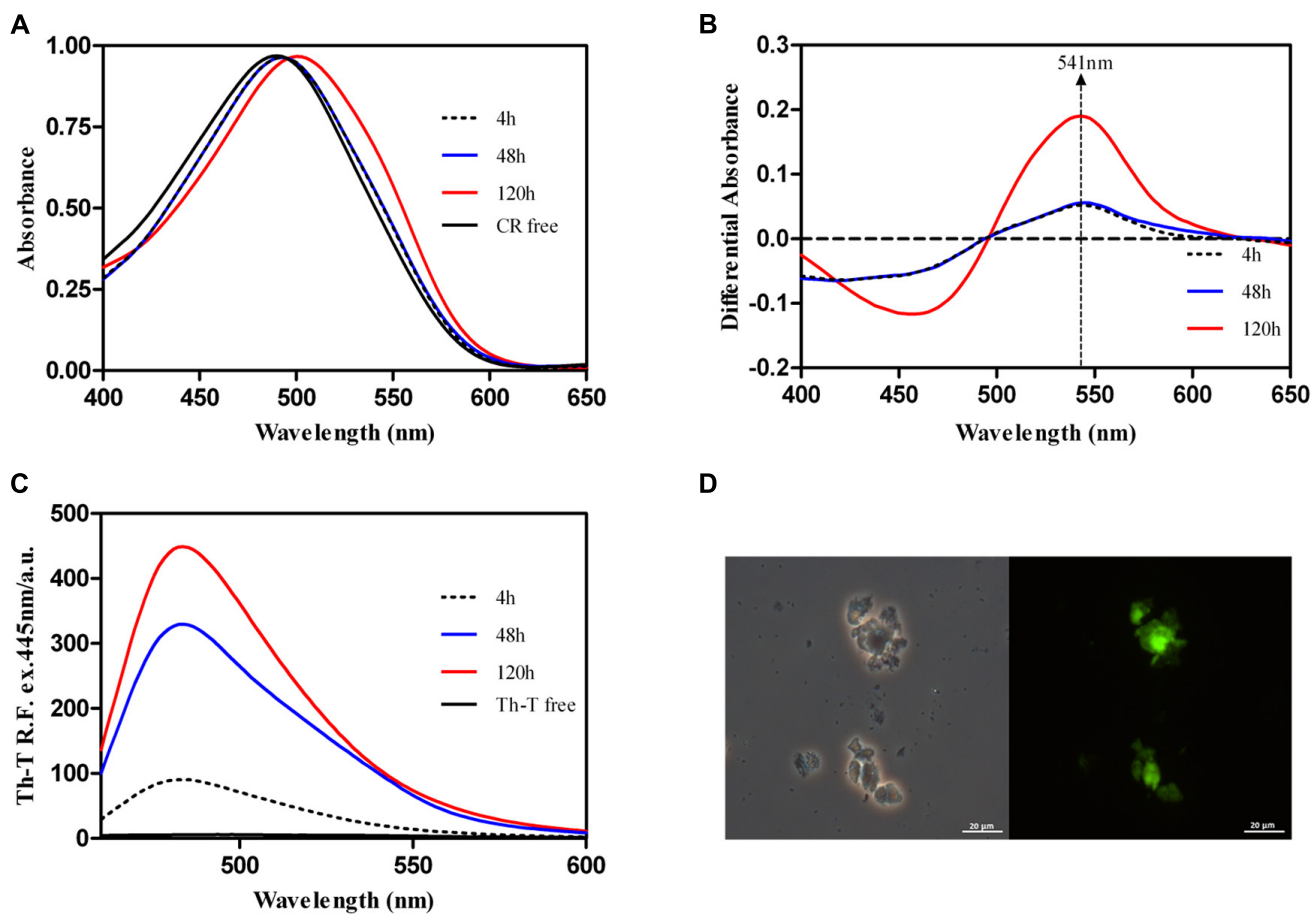
**Binding Rho cPrD aggregates to specific amyloid dyes. (A)** CR spectral changes in the presence of peptide incubated at different time **(B)** Difference absorbance spectra of CR displaying the characteristic amyloid band at 541 nm. **(C)** Fluorescence emission spectrum of Th-T when excited at 440 nm; note the characteristic fluorescence enhancement at ∼480 nm when the dye is bound to amyloid aggregates. **(D)** Rho cPrD peptide aggregates stained with Th-S and observed at 40X magnification by phase contrast and fluorescence microscopy displaying the green fluorescence characteristic of amyloid structures.

We used the amyloid-specific dyes CR, Th-T and Th-S to confirm that the detected β–sheet enriched aggregates were organized into amyloid-like suprastructures. The absorbance of CR increases and the spectrum maximum red shifts to 505–510 nm in the presence of peptide aggregates formed at 100 μM after 120 h of incubation at 25°C (**Figure 5A**). This spectral change corresponds to that promoted by different amyloid proteins in the aggregated state (Klunk et al., 1989). Moreover, the difference spectrum between the dye in the presence and absence of aggregated peptide allows detecting the characteristic amyloid band at ∼541 nm (**Figure 5B**). The binding of Rho cPrD to CR at early time points is significantly lower.

Thioflavin-T fluorescence emission is enhanced in the presence of amyloid fibrils (LeVine, 1993; Sabate et al., 2013). The same behavior is observed upon incubation of Th-T with Rho cPrD (**Figure 5C**). In good agreement with light scattering signals, Th-T binding to peptide solutions increases with incubation time, the Th-T fluorescence at the 480 nm spectral maximum increasing 80-fold at 120 h. Furthermore, binding of Th-S to 120 h aggregates could be visualized by fluorescence microscopy (**Figure 5D**). Areas rich in fibrous material were stained with Th-S to yield green–yellow fluorescence against a dark background.

**FIGURE 6:**
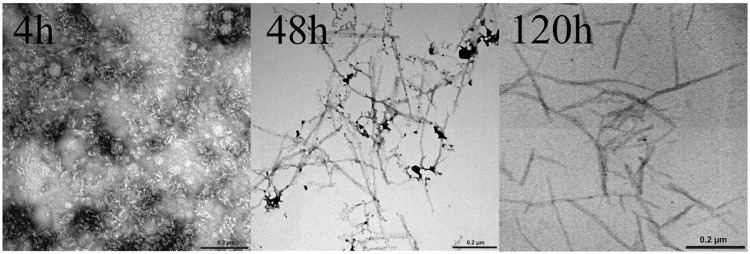
**Transmission electron micrographs of Rho cPrD after 4, 48, and 120 h of incubation at 25°C**.

The dye binding results indicate that incubated Rho cPrD solutions contain detectable amounts of amyloid-like structure. To confirm this extent, the morphological features of the peptide assemblies in these samples were analyzed using TEM. As shown in **Figure 6**, we detect the presence of protein aggregates in all cases. Nevertheless, in good agreement with spectroscopic data, the size and morphology of the aggregates are significantly different. The peptide incubated for 4 h forms short, poorly ordered protofibrilar assemblies. These assemblies coexist with fibrilar structures at the 48 h, whereas only mature fibrils with a typical amyloid-like morphology are observed at the 120 h.

**FIGURE 7:**
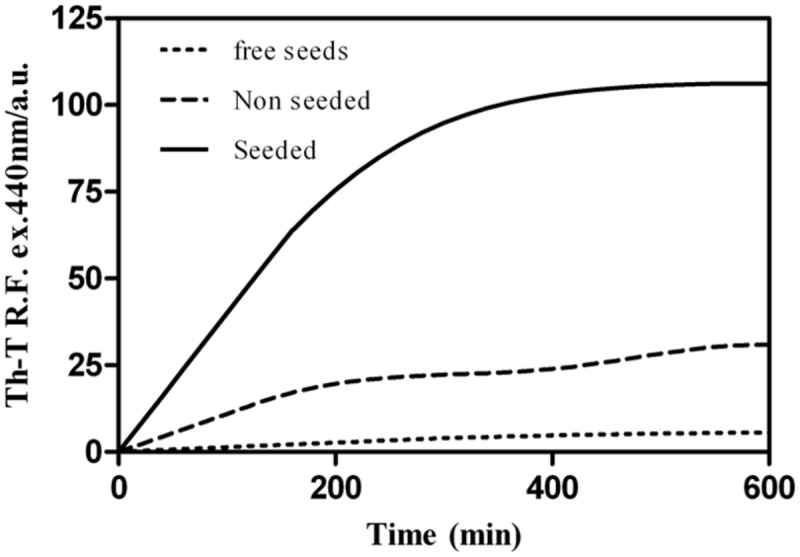
**Aggregation kinetics of Rho cPrD.** The aggregation reactions of 100 μM Rho cPrD were carried out under agitation at 25°C. 0.1 μM of *in vitro* formed fibrils (representing 0.1% of the final peptide concentration) were used for the seeding assay. The formation of Rho cPrD amyloid fibrils was accelerated in the presence of pre-aggregated peptide.

Seeded protein aggregation is a well-established mechanism for *in vivo* amyloid fibril formation and underlies prion propagation (Caughey, 2001; Wickner et al., 2001). The nucleation step of the amyloid assembly is shortened in the presence of preformed amyloid fibrils of the same protein that can act as nuclei for the subsequent polymerization reaction (Jarrett and Lansbury, 1992). Specific and short aggregation-prone regions have been shown to play a crucial role in this process (Pastor et al., 2007; Sabate et al., 2012). To test whether preformed Rho cPrD fibrils can seed the aggregation of the correspondent soluble peptide, we followed the aggregation kinetics of the peptide at 100 μM in the presence and absence of 0.1% (w/w) preformed fibrils. As shown in **Figure 7**, the presence of fibrils strongly accelerated the formation of Th-T positive assemblies, raising the possibility that such specific amyloid-promoting interactions could also occur in the context of the complete Rho factor protein.

## Discussion

Prion-like proteins were initially thought to be restricted to mammals, resulting in transmissible pathologies (Aguzzi and Weissmann, 1998). Later on, the discovery of yeast prions (Wickner, 1994; Du et al., 2008; Patel et al., 2009; Rogoza et al., 2010) and more recently of prion-like proteins in multicellular eukaryotes, from snail to human (Maji et al., 2009; Heinrich and Lindquist, 2011; Majumdar et al., 2012; Tariq et al., 2013; Cai and Chen, 2014), suggest that prion-like mechanisms would sustain evolutionary conserved functions in eukaryotic kingdoms. Despite no bacterial prion-like protein has been characterized so far, computational predictions support the existence of a significant number of proteins with potential prion-like properties in bacterial proteomes (Espinosa Angarica et al., 2014; Iglesias et al., 2015). This is not surprising, since bacterial cells have been shown to support the formation of prion-like conformations of yeast prions (Sabaté et al., 2009; Garrity et al., 2010; Espargaró et al., 2012) and, more importantly, to propagate them for over a hundred generations, even when the cells can no longer make the protein that serves as the trigger for the initial conversion (Yuan et al., 2014), which suggests that functional prion-like mechanisms might be more ancient than previously thought (Desantis et al., 2012).

As a trend, prion-like sequences are predicted to be less abundant in bacteria than in eukaryotes (Espinosa Angarica et al., 2013), but, interestingly, pathogenic species seem to have a higher prion load than non-pathogenic ones. An exciting possibility is that these sequences represent a bet-hedging mechanism for pathogens, as suggested recently for yeast prions (Newby and Lindquist, 2013). These mechanisms are used to diversify microbial phenotypes. In fluctuating environments this allows a fraction of the population to survive in conditions when most would perish. This mechanism would permit certain cells to persist in strenuous environments like in the presence of antibiotics or to escape the immunogenic response, saving the population from extinction. Shuﬄing the states of multiple prion-like proteins would allow rapid phenotypic diversification.

Here, we addressed the presence of potential prion-like proteins in the proteome of the pathogen *C. botulinum* using a stringent approach in which both a long region displaying amino acid compositional similitude to *bona fide* prions (Toombs et al., 2010, 2012) and the presence of a specific nucleating sequence inside it (Sabate et al., 2015b) should be present for a protein to be considered prion-like. This approach rendered a total of 54 candidates. Interestingly, the set of candidates is enriched in proteins that play a structural role and are linked to essential processes as cell wall metabolism or cellular shape maintenance. Although a more exhaustive analysis of these proteins is necessary, the data point to a possible relationship between the identified proteins and biofilm formation, which would confer a protecting strategy and facilitate the attachment of the bacteria to different surfaces. Indeed, the biofilms of a number of bacterial species have been shown to contain proteins in an amyloid conformation (Romero and Kolter, 2014) PrDs associated with proteins involved in survival and virulence were also found in *C. botulinum*. Sporulation and toxin production are powerful strategies that facilitate the invasion of new environments and bacterial survival in adverse conditions. In this context, proteins involved in spore formation and degradation, the degradation-protector NTNH in Botulism toxin and the cell-wall cross-linker L,D-transpeptidase develop non-essential functions, but facilitate the bacteria to remove toxic agents and evade the action of antibiotics or from harsh natural environmental conditions and toxic compounds (Biarrotte-Sorin et al., 2006; Magnet et al., 2007).

A significant number of the prion-like sequences predicted in the human proteome correspond to RNA binding proteins (King et al., 2012; Espinosa Angarica et al., 2013; Malinovska et al., 2013), which fits well with the fact that several experimentally determined *genuine* prion-like proteins, including Ure2, Swi1, Spf1, Cyc8, and Mot3 in yeast (Wickner, 1994; Du et al., 2008; Alberti et al., 2009; Patel et al., 2009; Rogoza et al., 2010) and *Drosophila melanogaster’*s GAGAfactor (Tariq et al., 2013) act as transcriptional regulators. This is also the function of the Rho factor in *C. botulinum*, for which we predict the existence of a highly scoring putative PrD at the N-terminus, adjacent to the RNA binding domain. It has been suggested that, in the prion-like state, transcriptional regulators may alter gene expression by creating diffusion barriers that restrict protein movement toward specific subcellular locations, by decreasing the effective concentration of the freely available pool of protein, or, on the contrary, by increasing the effective concentration in a certain location; this might result in enough functional diversity to create phenotypic divergence (Si, 2015). Interestingly enough, recent works have shown that Rho inhibition allows prophage maintenance, as a strategy to keep beneficial prophage genes, while silencing those likely to be deleterious (Cardinale et al., 2008; Menouni et al., 2013). Importantly, the pathogenic trait in *C. botulinum*, the botulinum neurotoxin, is mainly linked to a large plasmidome consisting of plasmids and circular prophages (Skarin and Segerman, 2014). Indeed, it has been recently shown that, in *E. coli*, mutations promoting adaptive properties, such us adaptation to thermal stress, converge to cluster either in the RNA polymerase complex or the termination factor Rho (Tenaillon et al., 2012; Rodriguez-Verdugo et al., 2014; Hug and Gaut, 2015). When we analyzed the mutations reported to occur specifically in Rho factor with our aggregation prediction algorithm AGGRESCAN (Conchillo-Solé et al., 2007), when found out that 72% of them endorse the terminator factor with increased aggregation-propensity, thus suggesting a link between the self-assembly of Rho and adaptation to changing environments.

We provide here strong evidence that detected PrD in Rho factor contains a short amyloid-like segment with the ability to potentially nucleate the Rho factor PrD assembly; however, it remains to be demonstrated if, in the case it occurs *in vivo*, the reaction would exhibit the reversibility required for considering this protein a *bona fide* prion.

In contrast to pWALTZ, conventional aggregation prediction algorithms do not capture the amyloidogenic potential of Rho cPrD. Because these latter algorithms usually display good accuracy when predicting the core of disease-linked amyloids (Sabate et al., 2015b), this suggests that the principles underlying their aggregation and that of Rho cPrD are somehow different. Indeed, the amyloid core of pathogenic proteins is usually very hydrophobic, whereas 90% of Rho cPrD sequence is made of N and S, and therefore polar, with only two hydrophobic residues. On the one hand, while a certain amyloid nucleation capacity favoring a sufficiently high aggregation rate is absolutely necessary, the final amyloid aggregate in a prion-like protein should at the same time display brittleness, a property that facilitates propagation. On the other hand, the protein should remain in a soluble state under physiological conditions, while keeping a cryptic amyloid capacity that allows it to self-assemble only in selected conditions. Both requirements imply that, in contrast to most amyloids, in PrDs, the aggregation reaction should not be nucleated by an extremely strong, and highly hydrophobic, amyloid core. We have proposed that the role of N residues in PrDs and their amyloid cores is to endorse these sequences with a basal aggregation propensity, while allowing them at the same time to remain soluble and disordered in normal cellular conditions (Sabate et al., 2015a; Zambrano et al., 2015). In contrast, the few hydrophobic residues found in these cores, especially aromatic ones, would play a key role in the initial amyloid oligomerization steps. This seems to be true for Rho cPrD since its assembly into amyloid-like fibrils is accompanied by an increase in the presence of hydrophobic clusters, as monitored by bis-ANS binding. It is very likely that, as described for amyloid peptides from the Sup35 prion (Balbirnie et al., 2001; Diaz-Avalos et al., 2003; van Der Wel et al., 2006; Zheng et al., 2006), complete hydrogen bonding of its N and S residues would also contribute to sustain the mature amyloid structure.

Aggregation constraints the evolution of proteins and accordingly nature have evolved different strategies to minimize protein aggregation in sequences and structures. Essentially, mutations that result in an increase in aggregation propensity tend to be purged out from the population, especially when they occur in a disordered context, since they are exposed to solvent, being this the reason that intrinsically disordered protein segments are in general, very soluble (Santner et al., 2012; Uversky, 2013, 2015; Graña-Montes et al., 2014). In this context, the inherent amyloid potential of Rho cPrD strongly suggests that this protein segment, and the surrounding predicted PrD, are conserved because they serve functional purposes in *C. botulinum*, in agreement with the general view that PrDs are important for protein–protein interactions and provide the flexibility required to self-organizing macromolecular assemblies in living cells (Malinovska et al., 2013; Iglesias et al., 2015).

## Conclusion

Overall, despite the reversibility and the functionality of *C. botulinum* Rho factor self-assembly should still be validated, this study provides a first proof for the existence of amyloidogenic sequences embedded in the recurrent putative PrD identified in transcription regulators of pathogenic bacteria, a property that is compatible with them being biological capacitors that might respond to environmental conditions rewiring gene expression.

## Author contributions

Conception/design of the work: SV, IP; performed the experiments: IP, VI; generated and analyzed the data: IP, VI, SV; drafting the work: IP, VI, SV; final approval of the manuscript to be published: SV, IP.

## Conflict of Interest Statement

The authors declare that the research was conducted in the absence of any commercial or financial relationships that could be construed as a potential conflict of interest.
